# Prognostic impact of p53 and/or NY‐ESO‐1 autoantibody induction in patients with gastroenterological cancers

**DOI:** 10.1002/ags3.12325

**Published:** 2020-03-25

**Authors:** Isamu Hoshino, Yoshihiro Nabeya, Nobuhiro Takiguchi, Hisashi Gunji, Fumitaka Ishige, Yosuke Iwatate, Fumiaki Shiratori, Satoshi Yajima, Rei Okada, Hideaki Shimada

**Affiliations:** ^1^ Division of Gastroenterological Surgery Chiba Cancer Center Chiba Japan; ^2^ Department of Hepatobiliary and Pancreatic Surgery Chiba Cancer Center Chiba Japan; ^3^ Department of Surgery School of Medicine Toho University Tokyo Japan

**Keywords:** esophageal squamous cell carcinoma, gastric cancer, hepatocellular carcinoma, NY‐ESO‐1, p53 gene

## Abstract

**Background and Aim:**

We evaluated the clinicopathological and prognostic significance of serum p53 (s‐p53‐Abs) and serum NY‐ESO‐1 autoantibodies (s‐NY‐ESO‐1‐Abs) in esophageal squamous cell carcinoma (ESCC), gastric cancer and hepatocellular carcinoma (HCC).

**Patients and Methods:**

A total of 377 patients, 85 patients with ESCC, 248 patients with gastric cancer, and 44 patients with HCC were enrolled to measure s‐p53‐Abs and s‐NY‐ESO‐1‐Abs titers by the enzyme‐linked immunosorbent assay before treatment. The clinicopathological significance and prognostic impact of the presence of autoantibodies were evaluated. Expression data based on the Cancer Genome Atlas and the prognostic impact of gene expression was also examined for discussion.

**Results:**

The positive rates of s‐p53‐Abs were 32.9% in ESCC, 15% in gastric cancer, and 4.5% in HCC. The positive rates of s‐NY‐ESO‐1‐Abs were 29.4% in ESCC, 9.7% in gastric cancer, and 13.6% in HCC. The presence of s‐p53‐Abs was not associated with tumor progression in these three cancer types. On the other hand, the presence of s‐NY‐ESO‐1‐Abs was significantly associated with tumor progression in ESCC and gastric cancer. The presence of s‐p53‐Abs and/or s‐NY‐ESO‐1‐Abs was significantly associated with poor prognosis in gastric cancer but not in ESCC nor HCC.

**Conclusions:**

The presence of s‐p53‐Abs and/or s‐NY‐ESO‐1‐Abs was associated with tumor progression in ESCC and gastric cancer. These autoantibodies might have poor prognostic impacts on gastric cancer (UMIN000014530).

## Graphical abstract

NY‐ESO‐1 antibodies were significantly associated with tumor progression in gastric cancer and esophageal squamous cell carcinoma, while the presence of p53 and/or NY‐ESO‐1 antibodies was significantly associated with poor prognosis only in gastric cancer.

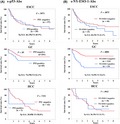

## 1 INTRODUCTION

The immunoglobulin G (IgG) autoantibodies against tumor antigens are known to appear in the serum of patients with cancer^1^ even in the early stages of tumor development. Therefore, such autoantibodies have potential as tumor markers for early detection.^1^, ^2^, ^3^ We have previously screened autoantibodies using the serological identification of antigens by recombinant expression cloning method and reported on their usefulness.^2^, ^4^ Among various autoantibodies, serum p53 antibodies (s‐p53‐Abs) and serum NY‐ESO‐1 antibodies (s‐NY‐ESO‐1‐Abs) are reported to show high positive rate in various cancer types.^3^, ^5^

The P53 gene is mutated in the majority of solid cancers, leading to the common expression of mutant p53 gene and protein in such diseases. s‐p53‐Abs appear in the cancer patients with this mutant p53 protein. In our previous studies, we also reported that s‐p53‐Abs are present in many cancer types.^3^, ^6^ In recent years, a high positive rate of s‐NY‐ESO‐1‐Abs was reported not only in esophageal cancer, but also in other cancer types.^5^, ^7^, ^8^ Although several reports showed clinicopathological significance of these two serum autoantibodies, prognostic impact has not been reported in the same patient group among gastroenterological cancers.

Therefore, we focused on the presence of s‐p53‐Abs and/or s‐NY‐ESO‐1‐Abs to examine the positive rate, clinicopathological significance, and prognostic impact on ESCC, gastric cancer, and HCC. Moreover, expression and prognostic impact of p53 and NY‐ESO‐1 gene expression were also discussed.

## 2 MATERIALS AND METHODS

### 2.1 Patients

The protocol was approved by the Ethics Committee of Chiba Cancer Center (no. 21‐26), and all patients provided written, informed consent. A total of 377 patients, 85 with ESCC, 248 with gastric cancer, and 44 with HCC, treated in Chiba Cancer Center between October 2008 and August 2010 were enrolled in this prospective study. The demographics of the patients are shown in Table 1.

**Table 1:** Patient details and clinicopathological features

	Esophageal cancer	Gastric cancer	Hepatocellular carcinoma
Number	85	248	44
Gender			
Male	73 (85.9)	181 (73.0%)	37 (84.1)
Female	12 (14.1)	67 (23.0)	7 (15.9)
Mean age ± s.d. (y)	68.2 ± 7.7	67.1 ± 10.5	63.4 ± 10.3
Age range (y)	45‐85	36‐89	46‐85
T‐classification			
T1	28 (32.9)	137 (55.2)	8 (18.2)
T2	8 (9.4)	32 (12.9)	15 (34.1)
T3	29 (34.1)	31 (12.5)	14 (31.8)
T4	20 (23.5)	48 (19.4)	7 (15.9)
Lymph node metastasis			
Positive	56 (65.9)	104 (41.9)	2 (4.5)
Negative	29 (34.1)	146 (58.1)	42 (95.5)
Distant metastasis			
Positive	19 (22.4)	47 (19.0)	5 (11.4)
Negative	66 (77.6)	201 (81.0)	39 (88.6)
TNM stage			
I	26 (30.6)	155 (62.5)	8 (18.2)
II	7 (8.2)	8 (3.2)	13 (29.5)
III	19 (22.4)	28 (11.3)	13 (29.5)
IV	33 (38.8)	57 (23.0)	10 (22.8)

Abbreviations: s.d., standard deviation.

### 2.2 Purification of recombinant p53 and NY‐ESO‐1 protein

For the expression and purification of recombinant protein, full‐length p53 (GenBank accession number AB082923) and NY‐ESO‐1 complementary DNA (NM 001327) were amplified via polymerase chain reaction. Other processing was performed according to an established protocol.^4^ DNA sequencing analysis was performed to confirm that the correct sequence was inserted into the constructed plasmid.

### 2.3 Detection of serum antibodies by enzyme‐linked immunosorbent assay

Serum samples from patients and healthy controls were analyzed via the enzyme‐linked immunosorbent assay, as previously described.^2^ The signals of s‐p53‐Abs and s‐NY‐ESO‐1‐Abs were evaluated by calculating the differences in absorbance between the wells containing antibodies and the wells containing phosphate‐buffered saline. The cut‐off values indicating positive reactivity were defined as optical density values greater than the mean values + 6 SD for s‐p53‐Abs and + 3 SD for s‐NY‐ESO‐1‐Abs for normal controls. The specificity of the assay was calculated according to the percentage of healthy controls from which negative results were obtained.

### 2.4 Gene expression analysis and survival analysis of p53 and NY‐ESO‐1

Aside from antibody expression, UALCAN (an interactive web portal for detailed analysis of TCGA gene expression data; available online: http://ualcan.path.uab.edu/) was used.^9^ The same web portal was used to analyze survival based on p53 and NY‐ESO‐1 gene expression level. Survival analysis with *P* < .05 was considered statistically significant. High‐expression patients show expression value >3rd quartile.

### 2.5 Statistical analysis

All analyses were performed using SPSS version 17.0 (SPSS, Inc.), Microsoft Excel (Microsoft), or GraphPad Prism (GraphPad Software). We performed a chi‐square test or Fisher's direct test to determine whether proportions of positive results differed significantly between patients with cancer and healthy controls and to correlate individual and complex antibody assay results with clinical parameters. The correlation between overall survival and autoantibody status was calculated using the log rank test, and the results are presented as a curve determined using the Kaplan‐Meier method. For all tests, *P*‐values <.05 (two‐tailed) were considered to indicate statistical significance.

## 3 RESULTS

### 3.1 Positive rate of P53 and NY‐ESO‐1 Abs in each carcinoma type

**Figure 1:**
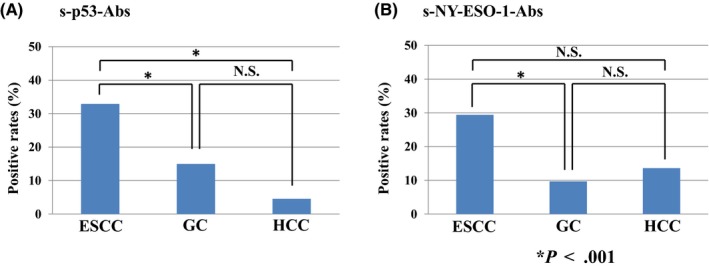
Positive rates of p53 and NY‐ESO‐1 antibodies in each cancer type

The positive rates of s‐p53‐Abs of each cancer were 32.9% in ESCC, 15% in gastric cancer, and 4.5% in HCC, respectively (Figure 1A). The positive rates of s‐NY‐ESO‐1‐Abs were 29.4% in ESCC, 9.7% in gastric cancer, and 13.6% in HCC, respectively (Figure 1B). The positive rates of s‐p53‐Abs and s‐NY‐ESO‐1‐Abs in ESCC were significantly higher than those in gastric cancer and HCC (*P* < .001).

**Figure 2:**
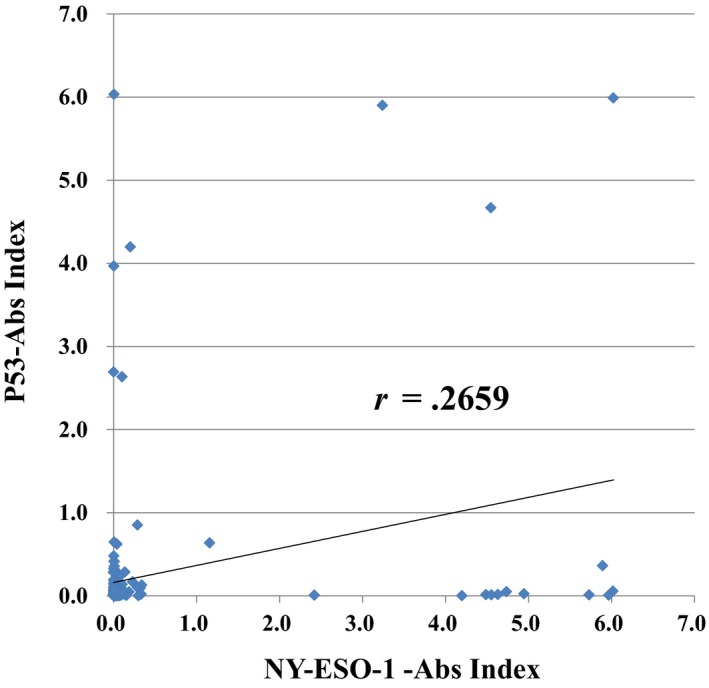
The Correlation between induction levels of p53 and NY‐ESO‐1

The relation between s‐p53‐Abs titers and s‐NY‐ESO‐1‐Abs titers were shown in Figure 2. There were no significant associations between s‐p53‐Abs titers and s‐NY‐ESO‐1‐Abs titers (*r* = .2659) (Figure 2).

### 3.2 Clinicopathological features and autoantibody status

In ESCC, the presence of s‐p53‐Abs was not associated with clinicopathological factors (Table 2). In gastric cancer, the presence of s‐p53‐Abs was significantly associated with men, lymph node metastases, and carcinoembryonic antigen. The presence of s‐NY‐ESO‐1‐Abs was also significantly associated with more advanced tumor invasion, distant metastasis, and advanced cases (Table 3). However, in HCC, s‐p53‐Abs and s‐NY‐ESO‐1‐Abs were not significantly associated with clinicopathological factors (Table 4).

**Table 2:** Patient details of panel positive in ESCC patients

	P53			NY‐ESO‐1		
Positive	−	+		−	+	
Number	57 (67.1%)	28 (32.9)		60 (90.3%)	25 (9.7)	
Gender						
Male (%)	49 (57.6)	24 (28.2)	*P* = .764	54 (63.4)	19 (22.4)	*P* = .178
Female	8 (9.4)	4 (4.7)		6 (7.1)	6 (7.1)	
Mean age ± s.d. (y)	68.8 ± 7.8	67.0 ± 7.2		67.7 ± 7.5	69.4 ± 8.0	
Age range (y)	45‐85	58‐82		45‐85	60‐84	
T‐classification						
T1	19 (22.4)	9 (10.6)	*P* = .912	25 (29.4)	3 (3.5)	***P* < .005**
T2	6 (7.1)	2 (2.4)		7 (8.2)	1 (1.2)	
T3	18 (21.2)	11 (12.9)		12 (14.1)	17 (20.0)	
T4	18 (21.2)	6 (7.1)		16 (18.8)	4 (4.7)	
Lymph node metastasis						
Positive	37 (43.5)	19 (22.4)	*P* = .979	35 (41.2)	21 (24.7)	***P* = .023**
Negative	20 (23.5)	9 (10.6)		25 (29.4)	4 (4.7)	
Distant metastasis						
Positive	11 (12.9)	8 (9.4)	*P* = .412	10 (11.8)	9 (10.6)	*P* = .096
Negative	46 (54.1)	20 (23.5)		50 (58.8)	16 (18.8)	
TNM stage						
I	19 (22.4)	7 (8.2)	*P* = .325	24 (28.2)	2 (2.4)	*P* = .063
II	2 (2.4)	5 (5.9)		4 (4.7)	3 (3.5)	
III	14 (16.5)	5 (5.9)		10 (11.8)	9 (10.6)	
IV	22 (25.9)	11 (12.9)		22 (25.9)	11 (12.9)	
CEA						
Positive	13 (15.3)	8 (9.4)	*P* = .755	15 (17.6)	6 (7.1)	*P* = .858
Negative	44 (51.8)	20 (23.5)		45 (52.9)	19 (22.4)	
SCC						
Positive	23 (27.1)	12 (14.1)	*P* = .825	22 (22.4)	13 (15.3)	*P* = .191
Negative	34 (40.0)	16 (18.8)		38 (67.1)	12 (14.1)	

Abbreviations: CEA, carcinoembryonic antigen; s.d., standard deviation. Factors with statistically significant differences are indicated by bold *P*‐values.

**Table 3:** Patient details of panel positive in Gastrc cancer patients

	P53			NY‐ESO‐1		
Positive	**‐**	+		‐	+	
Number	212 (85.5%)	36 (14.5)		224 (90.3%)	24 (9.7)	
Gender						
Male (%)	149 (60.1)	32 (12.9)	***P* = .034**	162 (65.3)	19 (7.7)	*P* = .634
Female	63 (25.4)	4 (1.6)		62 (25.0)	5 (2.0)	
Mean age ± s.d. (y)	67.3 ± 9.9	67.3 ± 10.2		67.1 ± 10.5	71.1 ± 8.4	
Age range (y)	37‐89	47‐84		37‐89	52‐81	
T‐classification						
T1	118 (47.6)	19 (7.7)	*P* = .939	130 (52.4)	7 (2.8)	***P* = .043**
T2	28 (11.3)	4 (1.6)		29 (11.7)	3 (1.2)	
T3	27 (10.9)	4 (1.6)		24 (9.7)	7 (2.8)	
T4	39 (15.7)	9 (3.6)		41 (4.8)	7 (2.8)	
Lymph node metastasis						
Positive	61 (24.6)	18 (7.3)	***P* = .011**	68 (27.4)	11 (4.4)	*P* = .122
Negative	151 (60.9)	18 (7.3)		156 (48.0)	13 (2.4)	
Distant metastasis						
Positive	40 (16.1)	7 (2.8)	*P* = .882	36 (14.5)	11 (4.4)	***P* < .001**
Negative	172 (69.4)	29 (11.7)		188 (75.8)	13 (5.3)	
Peritoneal dissemination						
Positive	25 (10.1)	6 (2.4)	*P* = .586	25 (10.1)	6 (2.4)	*P* = .104
Negative	187 (75.4)	30 (12.1)		199 (80.2)	18 (7.3)	
TNM stage						
I	137 (55.2)	18 (7.3)	*P* = .412	146 (58.9)	9 (3.6)	***P* = .019**
II	7 (2.8)	1 (0.4)		8 (3.2)	0 (0.0)	
III	21 (8.5)	7 (2.8)		26 (10.5)	2 (0.8)	
IV	47 (19.0)	10 (4.0)		45 (11.3)	12 (4.8)	
CEA						
Positive	32 (12.9)	11 (4.4)	***P* = .023**	35 (14.1)	8 (3.2)	*P* = .058
Negative	180 (72.6)	25 (10.0)		189 (76.2)	16 (6.5)	
CA19‐9						
Positive	28 (11.3)	7 (2.8)	*P* = .462	29 (11.7)	6 (2.4)	*P* = .192
Negative	184 (74.2)	29 (11.7)		195 (78.6)	18 (7.3)	

Abbreviations: CEA, carcinoembryonic antigen; s.d., standard deviation. Factors with statistically significant differences are indicated by bold *P*‐values.

**Table 4:** Patient details of panel positive in HCC patients

	P53			NY‐ESO‐1		
Positive	**‐**	**+**		**‐**	**+**	
Number	42 (95.5%)	2 (14.5)		38 (86.4%)	6 (13.6)	
Gender						
Male (%)	35 (79.6)	2 (4.5)	*P* = 1.000	33 (75.0)	4 (9.1)	*P* = .238
Female	7 (15.9)	0 (0.0)		5 (11.4)	2 (4.5)	
Mean age ± s.d. (y)	63.5 ± 10.0	61.5 ± 21.9		64.5 ± 10.2	56.3 ± 8.2	
Age range (y)	47‐85	46‐77		46‐85	48‐71	
T‐classification						
T1	8 (18.2)	0 (0.0)	*P* = .970	6 (13.6)	2 (4.5)	*P* = .626
T2	14 (31.8)	1 (2.3)		12 (27.3)	3 (6.8)	
T3	14 (31.8)	0 (0.0)		14 (31.8)	0 (0.0)	
T4	6 (13.6)	1 (2.3)		6 (13.6)	1 (2.3)	
Lymph node metastasis						
Positive	1 (2.3)	1 (2.3)	*P* = .090	1 (2.3)	1 (2.3)	*P* = .257
Negative	41 (93.1)	1 (2.3)		37 (84.1)	5 (11.4)	
Distant metastasis						
Positive	4 (9.1)	1 (2.3)	*P* = .217	4 (9.1)	1 (2.3)	*P* = .538
Negative	38 (86.4)	1 (2.3)		34 (77.3)	5 (11.4)	
TNM stage						
I	8 (18.2)	0 (0.0)	*P* = .457	6 (13.6)	2 (4.5)	*P* = .660
II	13 (29.5)	0 (0.0)		10 (22.7)	3 (6.8)	
III	13 (29.5)	0 (0.0)		13 (29.5)	0 (0.0)	
IV	8 (18.2)	2 (4.5)		9 (20.5)	1 (2.3)	
AFP						
Positive	27 (61.4)	1 (2.3)	*P* = 1.000	24 (54.5)	4 (9.1)	*P* = 1.000
Negative	15 (34.0)	1 (2.3)		14 (31.8)	2 (4.5)	
PIVKA‐II						
Positive	29 (65.9)	0 (0.0)	*P* = .111	26 (59.1)	3 (6.8)	*P* = .394
Negative	13 (29.5)	2 (4.5)		12 (27.3)	3 (6.8)	

### 3.3 Prognostic impact of autoantibodies

**Figure 3:**
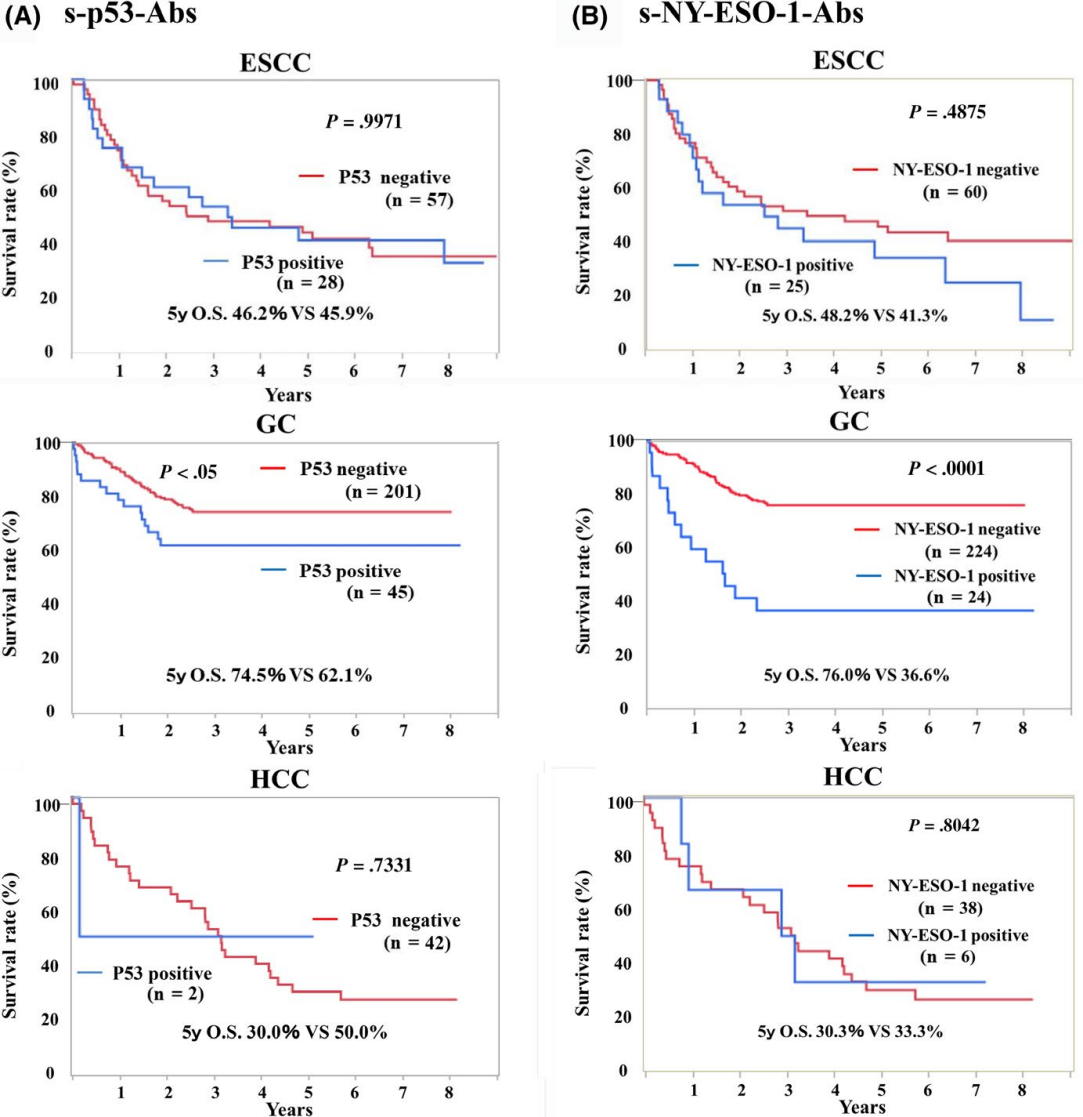
Prognostic role of autoantibodies in patients with various cancers

**Figure 4:**
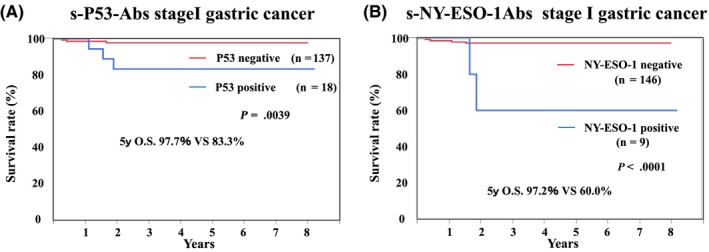
Prognostic role of autoantibodies in patients with cStageI GC

Overall survival rates for each cancer type were compared between the autoantibody‐positive and ‐negative groups (Figure 3). In ESCC, there was no significant difference between s‐p53‐Abs negative and positive groups (Figure 3A). Similarly, although s‐NY‐ESO‐1‐Abs negative group showed slightly better prognosis, the difference was not statistically significance (Figure 3B). In gastric cancer, the s‐p53‐Abs negative group showed significantly better prognosis than did the positive group (5‐year survival rate: negative vs positive group = 74.5% vs 62.1%, *P* < .05). Similarly, only in gastric cancer, the s‐NY‐ESO‐1‐Abs negative group showed significantly better survival than did the positive group (5‐year survival rate: negative vs positive group = 76.6% vs 36.6%, *P* < .0001). In addition, we extracted stage I gastric cancer patients and examined the prognosis of P53 or NY‐ESO‐1 antibody‐positive patients (Figure 4). As a result, in stage I, s‐p53‐Abs (*P* = .0039) and s‐NY‐ESO‐1‐Abs (*P* < .001) positive groups were shown to have significantly poorer prognosis compared to antibody negative group. In HCC, there were no significant differences between autoantibody positive and negative group, both for s‐p53‐Abs and s‐NY‐ESO‐1‐Abs (data not shown).

**Figure 5:**
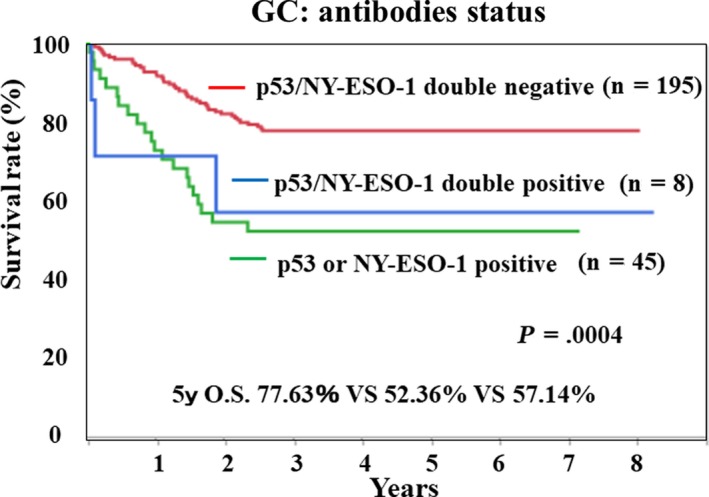
Prognostic analysis of p53 and/or NY‐ESO‐1 antibodies status

In gastric cancer, both antibodies resulted in poor prognosis in the antibody‐positive group. Therefore, the relationship between the inductions of each antibody was examined. There was no significant correlation between the titer of s‐p53‐Abs and s‐NY‐ESO‐1‐Abs (Figure 4). In addition, we compared the prognosis between three groups; s‐p53‐Abs and s‐NY‐ESO‐1‐Abs double negative group, single‐positive group, and double‐positive group. We found that there was a big difference in prognosis between double negative group and other groups. However, there was no difference in prognosis between either single‐positive group or double‐positive group (Figure 5).

## 4 DISCUSSION

In this study, we evaluated the positive rate, clinicopathological significance, and prognostic impact of s‐p53‐Abs and s‐NY‐ESO‐1‐Abs in ESCC, gastric cancer, and HCC. ESCC showed the highest positive rates for both autoantibodies. The presence of these autoantibodies was associated with tumor progression in ESCC and gastric cancer, but not in HCC. Both types of autoantibodies were correlated with prognosis in gastric cancer, but not in ESCC or HCC.

In this study, the positive rate of autoantibodies was highest in ESCC. On the other hand, we found that the presence of autoantibodies was associated with poor overall survival only in gastric cancer. We could not confirm the prognostic impact of s‐p53‐Abs in ESCC previously reported by Suzuki et al.^10^ The difference can be partly explained by different cut‐off values and/or different assay systems. In the present study, s‐NY‐ESO‐1‐Abs showed constantly high positive rates in the three types of gastroenterological cancers. The presence of s‐NY‐ESO‐1‐Abs was also found to be a predictor of poor prognosis in gastric cancer. Based on these results, additional treatment might be necessary for patients who are positive for these antibodies. However, in order to prove it, it is necessary to conduct a large‐scale detailed study such as multicenter research.

It has been suggested that the process resulting in antibody induction after gene alteration is complicated. For example, although p53 gene mutations are found in many patients with cancer (50%‐90%), only approximately half of these patients actually become positive for antibodies.^11^ A previous systematic review generally describes a moderate relationship between the frequency of p53 gene mutations and p53 antibody expression.^12^However, ESCC, head and neck, and colorectal cancer have relatively high p53 mutation rates and high antibody expression rates, while prostate cancer, glioma, and skin cancer have relatively high mutation rates and low antibody expression rates.^12^ Therefore, the relationship between gene mutation and antibody expression differs depending on cancer type. The autoantibody induction process involves various mechanisms and is affected by the expression and structure of the antigen protein and the immune response system.

Using the Cancer Genome Atlas (TCGA, available online: https://cancergenome.nih.gov/)^13^ database, the prognostic impact of p53 and NY‐ESO‐1 gene expression on esophageal squamous cell carcinoma (ESCC), gastric cancer, and hepatocellular carcinoma (HCC) can be evaluated. We found that except for the expression of NY‐ESO‐1 in ESCC, p53 and NY‐ESO‐1 gene expression levels were higher in patients with all cancer types than in healthy subjects (Figure S1). Notably, for NY‐ESO‐1, there is no correlation between protein expression in cancer tissue and serum antibody levels.^12^ In addition, the NY‐ESO‐1 low‐expression group tended to have a better prognosis than did the high‐expression group in HCC (Figure S2B). On the other hand, no association was found between gene expression and prognosis in gastric cancer or ESCC. On the other hand, gene expression only in HCC was significantly associated with prognosis. Actually, the gene expression data were not based on the same groups as were the serum autoantibody analysis data in this present study.

Limitations of this study were the small number of included cases and that all cases originated at a single institution. In particular, prognostic evaluation was difficult for HCC, because the antibody‐positive rate was the lowest and the number of cases was also the lowest among the examined cancer types. In addition, since serum sample was collected only once at the first consultation, the antibody titer over time could not be evaluated. For this reason, we have not been able to examine changes in antibody titers due to cancer progression or treatment effects.

To demonstrate these results, it may be necessary to conduct a larger sample size and collect samples multiple times, for example, to conduct clinical trials in collaboration with other institutions.

Regarding the discussion on gene expression analysis, TCGA data is mainly based on data from overseas, and most of the data is from Caucasians. It is necessary to examine gene expression in the same patient at the same time for accurate comparison with the expression data, however, there is no remaining sample and it cannot be demonstrated. In the future, we would like to consider testing with samples from the same patient.

In this study, s‐p53‐Abs and s‐NY‐ESO‐1‐Abs were evaluated on the same cohort for clinicopathological and prognostic impact in ESCC, gastric cancer, and HCC. Although s‐p53‐Abs and s‐NY‐ESO‐1‐Abs were significantly associated with tumor progression, the presence of these antibodies predicted poor prognosis only in gastric cancer.

## DISCLOSURE

Conflict of interest: Hideaki Shimada received research grants and technical lecture fees from the Medical & Biological Laboratories Co., Ltd. (Nagoya, Japan). The other authors declare no conflicts of interest in association with the present study.

## Supporting information

Figure S1

Figure S2
