# Supplementary figures and images for: Prognostic impact of p53 and/or NY‐ESO‐1 autoantibody induction in patients with gastroenterological cancers
Source: Ann Gastroenterol Surg. 2020 Mar 25;4(3):275–82. doi: 10.1002/ags3.12325 (PMC7240143; doi:10.1002/ags3.12325)

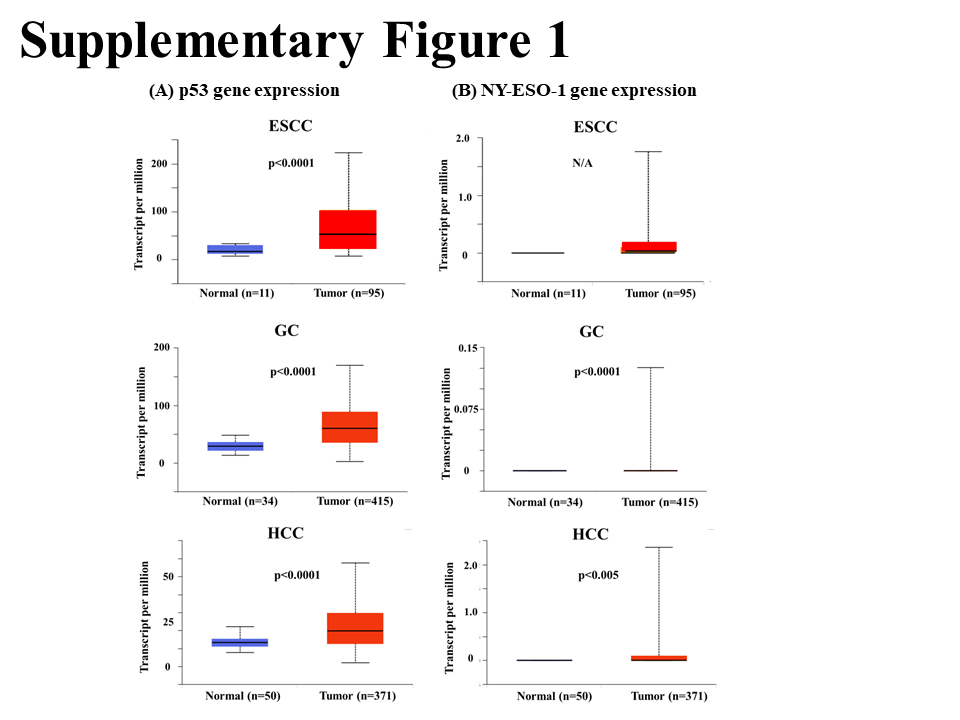

Supplement: Supplementary file 1 — Figure S1 [file AGS3-4-275-s001.tif]

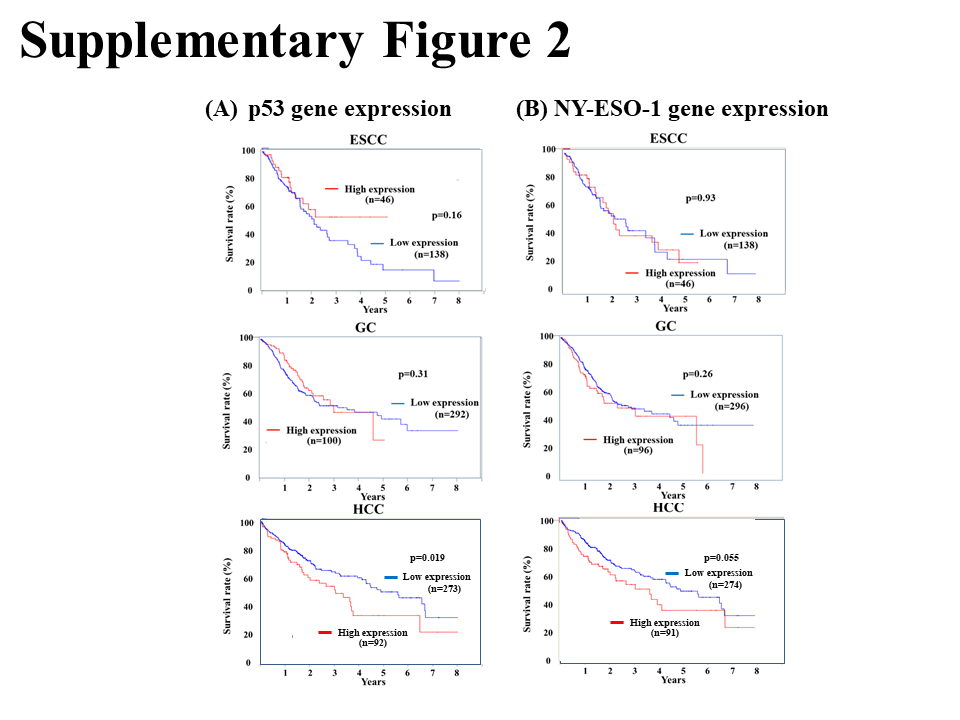

Supplement: Supplementary file 2 — Figure S2 [file AGS3-4-275-s002.tif]
